# Tratamento endovascular versus tratamento aberto de aneurisma de artéria poplítea: artigo de revisão

**DOI:** 10.1590/1677-5449.004917

**Published:** 2018

**Authors:** Rodrigo Nóbrega Bandeira, Daniel Guimarães Cacione, Francisco Chavier Vieira Bandeira, Ariane de Sousa Pelissoni, Cibele Ohany Nogueira Leite, Luis Carlos Uta Nakano

**Affiliations:** 1 Universidade Federal da Paraíba – UFPB, Departamento de Cirurgia, João Pessoa, PB, Brasil.; 2 Universidade Federal de São Paulo – UNIFESP, Departamento de Cirurgia Vascular e Endovascular, São Paulo, SP, Brasil.; 3 Universidade Federal de Juiz de Fora – UFJF, Departamento de Cirurgia, Juiz de Fora, MG, Brasil.; 4 Faculdade de Medicina Nova Esperança – FAMENE, Departamento de Cirurgia, João Pessoa, PB, Brasil.

**Keywords:** aneurisma, artéria poplítea, procedimentos endovasculares

## Abstract

O tratamento convencional do aneurisma da artéria poplítea é a cirurgia aberta de exclusão do aneurisma e revascularização do membro acometido. Nos últimos anos, o tratamento endovascular vem ganhando popularidade e interesse. O tratamento endovascular é menos invasivo e de menor morbidade; porém, é de alto custo e sua perviedade é incerta. O objetivo desta revisão é comparar os dois tratamentos através da análise de desfechos abordados em estudos primários e secundários. Realizou-se uma revisão narrativa da literatura publicada nos últimos 5 anos. Foram selecionados seis estudos retrospectivos, duas metanálises, um ensaio clínico e uma revisão sistemática Cochrane. Número limitado de pacientes e curto período de seguimento não nos permitem extrair conclusões consistentes. Não há evidência clara que sugere melhores resultados entre um ou outro tratamento eletivo. Novos ensaios randomizados devem ser realizados para determinar o papel do tratamento endovascular desse aneurisma.

## INTRODUÇÃO

 O aneurisma da artéria poplítea (AAP) é definido como uma dilatação focal maior que 50% do diâmetro máximo esperado para o segmento, considerando sexo e idade 1 , medido através da ultrassonografia, tomografia computadorizada ou ressonância nuclear magnética. A artéria poplítea é o segundo local mais frequente de aneurismas arteriais 2 . Suas complicações incluem: ruptura, embolização, trombose e perda do membro 3 . 

 O AAP ocorre frequentemente de forma bilateral. Em 40 a 60% dos indivíduos, a doença aneurismática também é observada em outros níveis. Sua incidência é menor que 0,1%; porém, representa 70% dos aneurismas periféricos, afetando principalmente pessoas com idade maior que 65 anos. Sua prevalência em homens entre 60-80 anos é estimada em 1% 4 ^,^ 5 e afeta cerca de 20 homens para cada mulher 6 . Na maioria dos indivíduos, o AAP é assintomático; entretanto, em 30% dos casos pode trazer complicações, especialmente embolização ou trombose do aneurisma 7 . 

 Pacientes assintomáticos, com aneurismas de diâmetro menor que 2 cm e sem trombos são mantidos em acompanhamento periódico com ultrassom com Doppler 8 ^,^ 9 . Indicações para o tratamento cirúrgico não são bem definidas. Porém, os critérios mais aceitos incluem: diâmetro maior que 2 cm, especialmente na presença de trombo mural, e casos sintomáticos 10 . 

 O tratamento aberto (TA) já mostrou ser extremamente durável, com excelente perviedade a longo prazo (perviedade primária superior a 76% em 5 anos) 11 ^,^ 12 , tendo como preferência a ponte (*by-pass*) com abordagem medial, ligadura proximal e distal do aneurisma e enxerto de veia safena magna invertida 13 . 

 Em 1994, Marin et al. 14 relataram o primeiro caso de tratamento endovascular (TE) de AAP. Esse tratamento promissor é menos invasivo, possui menor tempo cirúrgico e menor perda sanguínea, além de poder ser realizado com anestesia local e, consequentemente, propiciar alta hospitalar e recuperação precoces. 

 O TE do AAP é um tema polêmico e que gera controvérsias. Nos últimos anos, várias publicações tentaram avaliar a eficácia e a segurança desse tratamento em comparação ao TA do AAP. O objetivo desta revisão narrativa é comparar os dois tipos de tratamento através da análise de estudos primários ou secundários que contemplem o tema, realizando análise dos desfechos abordados. 

## MÉTODOS

 Realizou-se uma pesquisa bibliográfica nas bases de dados PubMed, SciELO e LILACS entre 2012 e 2017, utilizando os termos *aneurysm*, *popliteal artery* e *endovascular procedures*. Foram selecionados nove artigos que comparavam o TA com o TE: seis estudos retrospectivos, duas metanálises e um estudo em desenvolvimento. Foi adicionada também uma recente revisão sistemática da Cochrane. 

## RESULTADOS

 Os seis artigos de estudos retrospectivos utilizados nesta revisão foram os de: Pulli et al. 15 , Huang et al. 16 , Serrano Hernando et al. 17 , Ronchey et al. 18 , Cervin et al. 19 e Braga et al. 13 , os quais se encontram resumidos nas Tabelas 1 e 2 . 

**Tabela 1:** Estudos retrospectivos utilizados nesta revisão.

**Trabalho**	**Período**	**Aneurismas**		**Perviedade primária em %** **(1/2/3 anos)**			**Perviedade secundária em %** **(1/2/3 anos)**			**Endoprótese utilizada**	**Seguimento médio**	**Sucesso técnico em %**	**Vasos de escoamento**
		TA	TE	TA		TE	TA		TE				
				BV	BP		BV	BP					
Pulli et al. 15	2000~2011	178	134	78,8/77,1/-		79,1/76,9/-	84,7/82,7/-		84,7/82,7/-	Hemobahn® ou Viabahn®	27 m TA 35 m TE	100	< 2 v 39% TA 26% TE
Huang et al. 16	2005~2012	107	42	-/-/77 Em -/-/85 El		-/-/54 Em -/-/75 El	-/-/84 Em -/-/93 El		-/-/79 Em -/-/83 El	Viabahn®	3,8 a TA 2,6 a TE	98	0/1/2/3 v: TA (%): 2/31/45/21 TE (%): 4/25/38/32
Serrano Hernando et al. 17 *	1993~2013	139	32	94,9/94,9/-	83,3/74,9/-	79,7/79,7/-	100/100/-	93,3/80,3/-	89,3/88,3/89,3	Hemobahn® ou Viabahn®	49 m TA 22 m TE		0/1 v: 33,8% TA e 18,7% TE 2/3 v: 66,2% TA e 81,2% TE
Ronchey et al. 18 *	2000~2013	67 pacientes								Viabahn®		100	Em média 2,2 TE, 1,8 BV, 2,3 BP
Cervin et al. 19 *	2008~2012	592											
Braga et al. 13 *	2008~2013	21	10	75/-/-		80/-/-				Viabahn®		90	≥ 2 para TE

TA = tratamento aberto; TE = tratamento endovascular; BV = *by-pass* venoso; BP = *by-pass* com PTFE; m = meses; a = anos; v = vasos; Em = emergência; El = eletivo.

* Dados ausentes não foram encontrados nos artigos.

**Tabela 2:** Dados demográficos e características clínicas.

**Trabalho**	**Idade média** **(em anos)**			**Sexo masculino**		**Diâmetro médio** **(em mm)**		**Stents** **(em média)**	**Comorbidades**									
									**Tabagismo**		**Coronariopatia**		**HA**		**DPOC**		**Dislipidemia**	
	**TA**		**TE**	**TA**	**TE**	**TA**	**TE**		**TA**	**TE**	**TA**	**TE**	**TA**	**TE**	**TA**	**TE**	**TA**	**TE**
Pulli et al. 15	70±8,9		74,9±7,9	13 7,5%	13 9,5%	33,5	34,7	2	98 55%	94 70%	61 34%	41 30,5%	142 80%	93 70%	45 25%	31 23%	60 33,5%	46 34%
Huang et al. 16 *	71±9,6		81±6,5	90 99%	35 100%	32±1,5	30±0,9	1-4			8 9%	12 34%	28 80%	65 71%	13 14%	8 23%	67 74%	28 80%
Serrano Hernando et al. 17 *	68,7		74,3	139 100%	32 100%	26,1	28,4		82 58,9%	17 53,1%	24 17,2%	8 25%	74 53,2%	16 50%	23 16,5%	4 12,5%	54 38,8%	11 34,4%
Ronchey et al. 18	BV BP	66±10 68±7	71 ± 6	26/92% 14/100%	23 92%	27 26	24	1,44	15/53% 9/64%	14 60%	14/50% 4/28%	10 40%	26/92% 14/100%	23 92%	5/17% 1/7%	11 44%	14/29% 4/64%	10 36%
Cervin et al. 19 *	70 (42-102)			95%					79%				68%					
Braga et al. 13 *	70,95		67,6	18 100%	9 100%			1,6	68,41%	99%	10,52%	33%	73,68%	88%			26,31%	66%

TA = tratamento aberto; TE = tratamento endovascular; BV = *by-pass* venoso; BP = by-pass com PTFE; HA = hipertensão arterial; DPOC = doença pulmonar obstrutiva crônica.

* Dados ausentes não foram encontrados nos artigos.

 As metanálises utilizadas foram as de von Stumm et al. 20 , publicada em 2015, envolvendo um total de 652 casos, e de Leake et al. 21 , publicada em 2017, envolvendo 14 estudos e 4.880 tratamentos de AAP. 

 Na revisão sistemática Cochrane 22 , publicada em 2014, realizou-se uma busca por ensaios controlados e randomizados comparando os dois tipos de tratamento do AAP. 

 O estudo OVERPAR 23 (“*Open versus Endovascular Repair of Popliteal Artery Aneurysm* ”) é um estudo prospectivo, randomizado, multicêntrico em desenvolvimento. 

## DISCUSSÃO

 O tratamento cirúrgico aberto, descrito por Edwards et al. 24 no final da década de 1960, que consiste na exclusão do AAP por ligação e revascularização com *by-pass* venoso, continua sendo o procedimento mais usado, pois promove resultados bastante satisfatórios, especialmente em casos eletivos, alcançando altas taxas de perviedade do enxerto e de salvamento do membro durante o seguimento 2 ^,^ 11 . 

 O TE, descrito inicialmente por Marin et al. 14 em 1994, tem emergido como uma alternativa possível em relação ao tratamento cirúrgico aberto, particularmente em pacientes de alto risco cirúrgico 25 ^,^ 26 . 

 Em 2013, Pulli et al. 15 publicaram um grande registro retrospectivo multicêntrico de sete hospitais italianos, avaliando 178 TA e 134 TE de AAP. Dos TA, 13 foram por aneurismectomia e interposição de enxerto (11 com prótese e dois com veia safena), em 73 o enxerto foi colocado por dentro do aneurisma (25 com prótese e 48 com veia safena), e em 92 foi realizada ligação proximal e distal do aneurisma com *by-pass* por enxerto (76 com prótese e 16 com veia safena). 

 O TE estava indicado se houvesse pelo menos 2 cm de colo proximal e distal, com perviedade de pelo menos um vaso. O TA foi preferido em pacientes sintomáticos, com anatomia complexa e frequentemente na presença de isquemia. Não houve diferença entre os grupos em relação ao gênero, fatores de risco e comorbidades. O tempo médio de seguimento foi de 27 meses para o TA e 35 meses para o TE. 

 Os resultados do TA permanecem bons tanto em pacientes sintomáticos quanto em assintomáticos. O enxerto com veia autóloga é superior ao enxerto com politetrafluoretileno (PTFE) em termos de resultados iniciais e a longo prazo (perviedade primária em 4 anos foi de 86,3% para veia autóloga e 56,3% para prótese), sendo considerado o material de escolha no TA 3 ^,^ 27 ^,^ 28 . Foram encontrados resultados melhores com a abordagem posterior, em concordância com os resultados dos centros participantes 11 . 

 Em conclusão, se uma análise meticulosa de cada lesão/paciente for realizada para escolher a terapêutica apropriada, resultados iniciais e a longo prazo são satisfatórios. Contudo, faz-se necessário um ensaio randomizado e controlado comparando os dois tratamentos. 

 Huang et al. 16 publicaram, em 2014, um estudo retrospectivo envolvendo 149 AAP, sendo 107 tratadas por TA e 42 por TE realizados na clinica Mayo, Minnesota, EUA. O TA foi realizado por endoaneurismorrafia, ligação proximal e distal ou extração do aneurisma, sendo 86 por enxerto com veia autóloga e 21 por enxerto com PTFE. Para o TE, o colo proximal e distal considerado foi de 1,5 cm e pelo menos um vaso distal pérvio. Foram desconsiderados para o TE pacientes com idade menor que 50 anos, pobre escoamento distal, sintomas compressivos ou que flexionavam o joelho mais que 90 graus. Pacientes eleitos para o TE tinham em média 10 anos a mais e comorbidades cardíacas mais frequentes. Não houve diferença entre os grupos em relação ao tamanho do aneurisma, distribuição dos sintomas e escoamento distal. TE nos pacientes em estado de emergência (ruptura ou isquemia aguda) resultaram em maior tempo de internação, 20% de mortalidade e 20% de taxa de amputação. Complicações prévias e trombose do enxerto, intervenções prévias e tardias, eventos adversos foram significantemente mais frequentes após TE de emergência, resultando em uma perviedade primária menor (54%) após 3 anos. O estudo falhou em provar a superioridade do TE em relação ao TA. Para o tratamento eletivo, TE teve resultados inferiores, pois houve uma maior tendência de acontecimento de eventos adversos e um maior número de reintervenções. Contudo, a perviedade em 3 anos foi similar após TA e TE. Porém, se a anatomia é favorável, o TE eletivo em pacientes idosos e de alto risco parece ser justificado. Em casos de emergência, TE e TA foram equivalentes. Eventos adversos foram igualmente frequentes e o TE não mudou o prognóstico da isquemia aguda do membro. O estudo reconhece a necessidade de um ensaio multicêntrico randomizado para AAP em pacientes com quadro agudo. 

 O estudo retrospectivo de Serrano Hernando et al. 17 analisou o tratamento do AAP, sendo 139 por TA e 32 por TE, entre 1993 e 2013. O TA foi realizado por ligação proximal e distal do aneurisma, *by-pass* venoso em 99 pacientes e PTFE em 40 pacientes. O TE foi escolhido para pacientes de alto risco cirúrgico, sem veia adequada para *by-pass* e com anatomia favorável (definido como colo proximal e distal maior que 10 mm e diferença de calibre entre os dois segmentos ≤ 2 mm). Não houve diferença significativa entre as perviedades primária e secundária dos grupos com tratamento convencional *versus* endovascular, sintomático *versus* assintomático, anastomose tibial *versus* poplítea. O *by-pass* venoso é o melhor tratamento de acordo com esse trabalho e com a literatura publicada. O TE pode ser uma opção viável em pacientes selecionados; contudo, seus benefícios reais ainda devem ser estabelecidos. 

 Ronchey et al. 18 , em estudo retrospectivo de 67 pacientes tratados para AAP, dividiu-os em três grupos: A para TE, B para *by-pass* com veia safena magna e C para enxerto com prótese. A perviedade primária para 5 anos nos grupos A, B e C foi, respectivamente, de 71%, 81% e 69%, sem diferença estatística entre os três grupos. A perviedade secundária para 5 anos para os três grupos foi, respectivamente, de 88%, 85% e 84%, sem diferença estatística entre os grupos. O TE foi escolhido para pacientes de alto risco, com anatomia favorável, colo proximal e distal de 1,5 cm e pelo menos um vaso distal pérvio. Em conclusão, o TE do AAP tem limitações anatômicas relacionadas aos movimentos da artéria, mas não é inferior ao tratamento cirúrgico com enxerto de prótese. Esse tratamento reduz a permanência hospitalar do paciente e a necessidade de transfusão sanguínea. O enxerto com veia safena magna não trouxe melhores resultados em comparação com o enxerto de prótese. Porém, o estudo reconhece a necessidade de ensaios controlados e randomizados com resultados a longo prazo e critérios de inclusão apropriados para comparar as três opções de tratamento. 

 Em 2015, Cervin et al. 19 publicaram registro retrospectivo do Sweedvasc (Registro Vascular Sueco), que agrupou dados de 30 hospitais, entre 2008 e 2012, analisando 592 tratamentos de AAP. Esses tratamentos foram analisados entre os casos de emergência (174 com isquemia aguda, 13 com ruptura) e os casos eletivos (300 assintomáticos, 105 com outros sintomas). Nos pacientes com isquemia aguda, a perviedade primária e secundária em 1 ano foi, respectivamente, de 78,8% e 86,8% para o TA; 42,9% e 47,6% para o TE. Nos pacientes sintomáticos, a perviedade primária e secundária em 1 ano foi, respectivamente, de 81,1% e 86,5% para o TA; 57,1% e 85,7% para o TE. Nos pacientes assintomáticos, a perviedade primária e secundária em 1 ano foi, respectivamente, de: 89% e 93,5% para o TA; 67,4% e 83,7% para o TE. 

 Esse estudo revela uma importante diferença clínica entre os grupos de TA e TE, favorecendo o TA. Apesar desse achado ter sido esperado, a magnitude da diferença dos resultados, em particular entre aqueles tratados por isquemia aguda, foi inesperado e coloca em questão o uso de TE para AAP. 

 A experiência de 5 anos do Hospital das Clínicas de Ribeirão Preto foi exposta por Braga et al. 13 . O TE com stent revestido Viabahn® (média de 1,6 por paciente) foi indicado para pacientes de alto risco cirúrgico, com no mínimo dois vasos distais pérvios e colo proximal e distal de pelo menos 1 cm. Foi indicado TA para pacientes com isquemia aguda, claudicação limitante ou assintomáticos com diâmetro > 2 cm, sendo realizado por ponte femoropoplítea com uso de veia safena magna invertida e ligadura distal e proximal do aneurisma. 

 Foram realizados 10 TE e 21 TA em 28 pacientes. Não houve diferença estatística no tempo médio de internação e na sobrevida do membro entre as duas técnicas em 30 e 90 dias. Foi necessária uma reabordagem em 10% dos casos endovasculares e 14,28% do grupo aberto em 30 dias. Nos casos de TA, além de não importar a anatomia, foram avaliados pacientes com isquemia crítica, levando a um viés de inclusão e de resultado, o que impede a comparação dos resultados dos grupos. Assim, estudos prospectivos e controlados são necessários para avaliar o TE como procedimento alternativo nos casos de alto risco com anatomia favorável. 

 Von Stumm et al. 20 publicaram em 2015 uma metanálise analisando um ensaio randomizado controlado combinado com um estudo de coorte prospectivo e quatro estudos de coorte retrospectivo, envolvendo, ao total, 652 casos. Essa metanálise sugere que o tratamento endovascular do AAP pode ser um método seguro e eficiente para pacientes com anatomia favorável. Perviedade primária a médio prazo não diferiu entre o TE e o TA; porém, os resultados de reintervenção e trombose em 30 dias foram melhores para o grupo de TE. Atualmente, a qualidade da evidência para o TE é baixa, e outras pesquisas são absolutamente necessárias para recomendações baseadas em evidências no TE. 

 Leake et al. 21 , em 2017, realizaram uma metanálise de 14 estudos publicados entre 2005 e 2016, envolvendo 4.880 AAP, sendo 3.915 por TA e 1.210 por TE. As comorbidades dos pacientes foram similares nos dois grupos de tratamento. Complicações da ferida foram cinco vezes mais frequentes no TA. Tempo de permanência hospitalar foi significantemente menor no grupo para TE. A perviedade primária para 1 e 3 anos foi melhor no TA. Não houve diferença significativa na perviedade secundária para 1 e 3 anos entre os dois tratamentos. TE promove menor taxa de complicações e menor tempo de hospitalização, porém a perviedade primária é menor. Novos estudos revelando resultados a longo prazo são necessários. 

 Em 2014 a Cochrane Collaboration publicou uma revisão sistemática 22 comparando os dois tipos de tratamento do AAP. Apenas um ensaio randomizado controlado foi identificado conforme os critérios de inclusão. Nessa publicação, 30 AAP foram tratados (15 por TA e 15 por TE) e acompanhados por pelo menos 4 anos. O tempo médio de internação hospitalar (4,3 dias, contra 7,7 dias no TA) e de cirurgia (75,4 minutos, contra 195,3 minutos no TA) foi significantemente menor no grupo para TE. A perviedade primária em 1 ano foi de 100% para o TA e de 93,3% para o TE, porém foi similar para 4 anos. A maior limitação do estudo foi o reduzido número de casos. Atualmente, não há evidência clara que sugere melhores resultados entre um ou outro tratamento eletivo. Há necessidade de mais ensaios randomizados com amostras adequadas para determinar alguma vantagem do tratamento endovascular sobre o tratamento aberto do AAP. 

 Apesar de o tratamento cirúrgico aberto ser o mais aceito para o AAP, o TE vem ganhando popularidade e interesse, devido à maior facilidade e à menor agressividade com relação ao tratamento cirúrgico. Como consequência, um grande número de artigos vem sendo publicado recentemente 25 ^,^ 26 ^,^ 29 ^-^ 34 . 

 Contudo, até o momento, apenas relatos de curta duração de hospitalização em pacientes tratados por via endovascular foram publicados, nos poucos artigos comparando os dois tratamentos 29 ^-^ 34 . Esses estudos têm várias limitações: são estudos retrospectivos não randomizados, com grandes discrepâncias entre os grupos de TA e TE em termos de apresentação clínica do paciente, curto período de seguimento e pequena amostra, principalmente no grupo para TE. 

 O número limitado de pacientes coletado nessas comparações não nos permite extrair conclusões consistentes. Novos ensaios controlados e randomizados, com resultados a longo prazo e critérios de inclusão apropriados para comparar as três opções de tratamento, são necessários para sabermos as reais indicações e contraindicações do tratamento endovascular, bem como sua eficácia e segurança. 

 O estudo OVERPAR 23 (“Open versus Endovascular Repair of Popliteal Artery Aneurysm”) é um estudo prospectivo, randomizado, multicêntrico em desenvolvimento, que promete ser o maior estudo já realizado em pacientes com aneurisma de artéria poplítea assintomático. Esse estudo comparativo proverá evidência em nível 1 para guiar o tratamento de pacientes com AAP. 

## CONCLUSÃO

 O tratamento cirúrgico aberto no manejo do AAP sintomático ou assintomático acima de 2 cm é considerado a modalidade de tratamento mais tradicional e com bons índices de perviedade. O TE, por sua vez, veio inicialmente como tratamento alternativo ao aberto em pacientes com risco cirúrgico aumentado; porém, com o passar dos anos passou a ser aventado como primeira opção de tratamento. No entanto, não há evidência clara que sugere melhores resultados entre um ou outro tratamento eletivo, como demonstrado pela revisão sistemática da Cochrane, que tem nível de evidência moderado. Novos ensaios randomizados, com amostras adequadas, longo período de seguimento e critérios bem definidos, devem ser realizados para determinar o papel do TE no AAP. 
