# Supplementary material for: Tratamento endovascular versus tratamento aberto de aneurisma de artéria poplítea: artigo de revisão
Source: J Vasc Bras. 2018 Jan-Mar;17(1):34–41. [Article in Portuguese] doi: 10.1590/1677-5449.004917 (PMC5990263; doi:10.1590/1677-5449.004917)
Supplement: Translation

# Translation: Endovascular vs. open repair of popliteal artery aneurysm: review article

**Authors:** Rodrigo Nóbrega Bandeira, Daniel Guimarães Cacione, Francisco Chavier Vieira Bandeira, Ariane de Sousa Pelissoni, Cibele Ohany Nogueira Leite, Luis Carlos Uta Nakano

**Affiliations:** 1 Universidade Federal da Paraíba – UFPB, Departamento de Cirurgia, João Pessoa, PB, Brasil.; 2 Universidade Federal de São Paulo – UNIFESP, Departamento de Cirurgia Vascular e Endovascular, São Paulo, SP, Brasil.; 3 Universidade Federal de Juiz de Fora – UFJF, Departamento de Cirurgia, Juiz de Fora, MG, Brasil.; 4 Faculdade de Medicina Nova Esperança – FAMENE, Departamento de Cirurgia, João Pessoa, PB, Brasil.

**Keywords:** aneurysm, popliteal artery, endovascular procedures

## Abstract

The conventional treatment for a popliteal artery aneurysm is open surgery to exclude the aneurysm and revascularization of the affected limb. Over recent years, endovascular treatment has grown in popularity and has been attracting increased interest. Endovascular treatment is less invasive and associated with lower rates of morbidity, but it is expensive and patency is uncertain. The aim of this review is to compare these two treatments by analyzing the outcomes reported in primary and secondary studies. A narrative review is conducted of the literature published over the last 5 years. Six retrospective studies, two meta-analyses, one clinical trial and one Cochrane systematic review were selected. We were unable to draw firm conclusions because of small patient samples and short follow-up periods. There is no clear evidence to indicate that the outcomes of one or another elective treatment are superior. New randomized trials should be conducted to determine the role endovascular treatment has to play in management of this type of aneurysm.

## INTRODUCTION

 A popliteal artery aneurysm (PAA) is defined as a focal dilation that exceeds 50% of the segment’s maximum expected diameter (estimated considering age and sex) 1 measured using ultrasonography, computed tomography, or magnetic resonance imaging. The popliteal artery is the second most common site of arterial aneurysms. 2 Complications include rupture, embolization, thrombosis, and limb loss. 3

 Popliteal artery aneurysms are often bilateral. In 40 to 60% of patients, aneurysmal disease is also found at other levels. Incidence is less than 0.1%; but they account for 70% of peripheral aneurysms, primarily affecting people over the age of 65 years. Their prevalence in men aged 60-80 years is estimated at 1% 4 ^,^ 5 and around 20 men are affected for each woman. 6 In the majority of patients, PAA is asymptomatic, but in 30% of cases it can involve complications, particularly embolization or thrombosis of the aneurysm. 7

 Asymptomatic patients, with aneurysm diameters less than 2 cm and free from thrombi are managed with periodic examinations using Doppler ultrasound. 8 ^,^ 9 Indications for surgical treatment have not been well-defined, but the most accepted criteria include diameter greater than 2 cm, especially in the presence of mural thrombus, and symptomatic cases. 10

 Open treatment (OT) has proven extremely durable, with excellent patency over the long term (primary patency exceeding 76% at 5 years). 11 ^,^ 12 By-pass is the preferred technique, with a medial approach, proximal and distal ligature of the aneurysm, and inverted great saphenous vein graft. 13

 In 1994, Marin et al. 14 reported the first case of endovascular treatment (ET) for PAA. This promising treatment is less invasive, requires shorter duration surgery and involves less blood loss, and can also be performed with local anesthesia and, as a consequence, hospital discharge and recovery occur earlier. 

 Endovascular treatment for PAA is a controversial subject. Over recent years there have been several publications describing attempts to assess the efficacy and safety of this treatment for PAA compared with OT. The objective of this narrative review is to compare both types of treatment through an analysis of primary or secondary studies of the subject, analyzing the outcomes reported. 

## METHODS

 Bibliographic searches were run on the PubMed, SciELO, and LILACS databases for publications from 2012 to 2017, using the terms aneurysm, popliteal artery, and endovascular procedures. A total of nine articles that compared OT with ET were selected: six retrospective studies, two metanalyses and one ongoing trial. A recent Cochrane systematic review was also included. 

## RESULTS

 The six articles describing retrospective studies were published by Pulli et al., 15 Huang et al., 16 Serrano Hernando et al., 17 Ronchey et al., 18 Cervin et al., 19 and Braga et al., 13 and are summarized in Tables 1 and 2 . 

**Table 1:** Retrospective studies utilized in this review.

**Study**	**Period**	**Aneurysms**		**Primary patency in %** **(1/2/3-year)**			**Secondary patency in %** **(1/2/3-year)**			**Endoprostheses used**	**Mean follow-up**	**Technical success in %**	**Run-off vessels**
		OT	ET	OT		ET	OT		ET				
				VBP	PBP		VBP	PBP					
Pulli et al. 15	2000~2011	178	134	78.8/77.1/-		79.1/76.9/-	84.7/82.7/-		84.7/82.7/-	Hemobahn® or Viabahn®	27 m OT 35 m ET	100	< 2 v 39% OT 26% ET
Huang et al. 16	2005~2012	107	42	-/-/77 Em -/-/85 El		-/-/54 Em -/-/75 El	-/-/84 Em -/-/93 El		-/-/79 Em -/-/83 El	Viabahn®	3.8 yrs OT 2.6 yrs ET	98	0/1/2/3 v: OT (%): 2/31/45/21 ET (%): 4/25/38/32
Serrano Hernando et al. 17 *	1993~2013	139	32	94.9/94.9/-	83.3/74.9/-	79.7/79.7/-	100/100/-	93.3/80.3/-	89.3/88.3/89.3	Hemobahn® or Viabahn®	49 m OT 22 m ET		0/1 v: 33.8% OT and 18.7% ET 2/3 v: 66.2% OT and 81.2% ET
Ronchey et al. 18 *	2000~2013	67 patients								Viabahn®		100	Mean 2.2 ET, 1.8 VBP, 2.3 PBP
Cervin et al. 19 *	2008~2012	592											
Braga et al. 13 *	2008~2013	21	10	75/-/-		80/-/-				Viabahn®		90	≥ 2 for ET

OT = open treatment; ET = endovascular treatment; VBP = venous bypass; PBP = PTFE bypass; m = months; yrs = years; v = vessels; Em = emergency; El = elective

* Missing data were not found in the articles.

**Table 2:** Demographic data and clinical characteristics.

**Study**	**Mean age** **(in years)**			**Male**		**Mean diameter** **(in mm)**		**Stents** **(mean)**	**Comorbidities**									
									**Smoking**		**Heart disease**		**AH**		**COPD**		**Dyslipidemia**	
	**OT**		**ET**	**OT**	**ET**	**OT**	**ET**		**OT**	**ET**	**OT**	**ET**	**OT**	**ET**	**OT**	**ET**	**OT**	**ET**
Pulli et al. 15	70±8.9		74.9±7.9	13 7.5%	13 9.5%	33.5	34.7	2	98 55%	94 70%	61 34%	41 30.5%	142 80%	93 70%	45 25%	31 23%	60 33.5%	46 34%
Huang et al. 16 *	71±9.6		81±6.5	90 99%	35 100%	32±1.5	30±0.9	1-4			8 9%	12 34%	28 80%	65 71%	13 14%	8 23%	67 74%	28 80%
Serrano Hernando et al. 17 *	68.7		74.3	139 100%	32 100%	26.1	28.4		82 58.9%	17 53.1%	24 17.2%	8 25%	74 53.2%	16 50%	23 16.5%	4 12.5%	54 38.8%	11 34.4%
Ronchey et al. 18	VBP PBP	66±10 68±7	71 ± 6	26/92% 14/100%	23 92%	27 26	24	1.44	15/53% 9/64%	14 60%	14/50% 4/28%	10 40%	26/92% 14/100%	23 92%	5/17% 1/7%	11 44%	14/29% 4/64%	10 36%
Cervin et al. 19 *	70 (42-102)			95%					79%				68%					
Braga et al. 13 *	70.95		67.6	18 100%	9 100%			1.6	68.41%	99%	10.52%	33%	73.68%	88%			26.31%	66%

OT = open treatment; ET = endovascular treatment; VBP = venous by-pass; PBP = by-pass with PTFE graft; AH = arterial hypertension; COPD = chronic obstructive pulmonary disease.

* Missing data were not found in the articles.

 The metanalyses used were conducted by von Stumm et al., 20 published in 2015 and combining a total of 652 cases, and by Leake et al., 21 published in 2017, covering 14 studies and 4,880 PAA treatments. 

 The Cochrane systematic review 22 was published in 2014 and is based on a search for randomized, controlled trials comparing the two types of PAA treatment. 

 The OVERPAR study 23 (“*Open* vs. *Endovascular Repair of Popliteal Artery Aneurysm* ”) is an ongoing prospective, randomized, multicenter trial. 

## DISCUSSION

 Surgical open treatment was described by Edwards et al. 24 at the end of the 1960s and consists of exclusion of the PAA by ligature and revascularization with venous by-pass and it remains the most widely used procedure, because the results are highly satisfactory, especially in elective cases, with high rates of graft patency and limb salvage observed over the course of follow-up. 2 ^,^ 11

 In turn, ET was first described in 1994 by Marin et al. 14 and has emerged as a possible alternative to treatment with open surgery, particularly for patients with high surgical risk. 25 ^,^ 26

 In 2013, Pulli et al. 15 published an analysis of a multicenter retrospective registry comprising data from seven Italian hospitals in which they evaluated 178 OT and 134 ET cases of PAA. Thirteen of the OT were performed by aneurysmectomy and graft interposition (11 with prosthesis and two with a saphenous vein graft), while in 73 cases the graft was placed inside the aneurysm (25 prosthetic grafts and 48 saphenous vein grafts), and in 92 cases proximal and distal aneurysm ligature was followed by by-pass grafting (76 with prostheses and 16 with saphenous veins). 

 Endovascular treatment was indicated if there was at least 2 cm of proximal and distal neck and at least one vessel was patent. Open treatment was chosen for symptomatic patients and patients with complex anatomy and often in the presence of ischemia. There were no differences between groups in relation to sex, risk factors, or comorbidities. Mean follow-up time was 27 months for OT and 35 months for ET. 

 Open treatment still offers good results for both symptomatic and asymptomatic patients. Grafts with autologous veins are superior to polytetrafluoroethylene (PTFE) grafts in terms of initial and long term results (primary patency at 4 years was 86.3% for autologous vein grafts and 56.3% for the prosthesis) and veins are considered the material of choice for OT. 3 ^,^ 27 ^,^ 28 Better results were observed when a posterior approach was used, in agreement with the results of the participating centers. 11

 In conclusion, if a meticulous analysis of each aneurysm/patient is conducted to choose the appropriate treatment, initial and long-term results are satisfactory. However, a randomized and controlled trial comparing the two treatments is necessary. 

 In 2014, Huang et al. 16 published a retrospective study of 149 PAAs, 107 of which had been treated with OT and 42 treated by ET at the Mayo clinic in Minnesota, United States. Open treatment was conducted by endoaneurysmorrhaphy with proximal and distal ligature or removal of the aneurysm, and 86 aneurysms were repaired with autologous vein grafts and 21 with PTFE grafts. The proximal and distal neck size considered necessary for ET was 1.5 cm with at least on patent distal vessel. Criteria for rejecting patients for ET were age less than 50, poor distal run-off, compressive symptoms, and knee flexion greater than 90 degrees. Patients who had elective ET were a mean of 10 years older and had a higher frequency of cardiac comorbidities. There were no differences between the groups in terms of aneurysm size, distribution of symptoms, or distal run-off. Emergency patients managed with ET (for rupture or acute ischemia) had longer hospital stays, 20% mortality, and a 20% amputation rate. Prior complications and thrombosis of the graft, prior and late interventions, and adverse events were all significantly more frequent after emergency ET, which had lower 3-year primary patency (54%). The study failed to prove the superiority of ET with relation to OT. Furthermore, ET had inferior results after elective treatment, since there was a trend to a higher rate of adverse events and a higher number of reinterventions. Nevertheless, 3-year patency was similar after OT and ET. Notwithstanding, if anatomy is favorable, then elective ET appears to be justified in high-risk elderly patients, while in emergency cases ET and OT were equivalent. Adverse events had equal frequency and ET did not change the prognosis of acute limb ischemia. This study recognized the need for a randomized multicenter study of PAA in patients with acute presentations. 

 The retrospective study by Serrano Hernando et al. 17 analyzed PAA treatments performed from 1993 to 2013, 139 with OT and 32 with ET. Open treatment was by proximal and distal ligation of the aneurysm, with venous by-pass in 99 patients and PTFE grafts in 40 patients. Endovascular treatment was chosen for patients with high surgical risk, without an adequate vein for by-pass, and with favorable anatomy (defined as proximal and distal necks greater than 10 mm and difference in caliber between the two segments ≤ 2 mm). There were no significant differences between primary or secondary patency rates when analyzed by conventional vs. endovascular treatment, symptomatic vs. asymptomatic, or tibial vs. popliteal anastomosis. Venous by-pass is the best treatment according to this study and to the published literature. Endovascular treatment may be a viable option in selected patients; but its true benefits have not yet been established. 

 Ronchey et al. 18 conducted a retrospective study of 67 PAA patients, dividing them into three groups: A for ET, B for by-pass with the great saphenous vein, and C for prosthetic grafting. Five-year primary patency rates for groups A, B, and C were 71%, 81%, and 69% respectively, with no statistical difference between the three groups. Five-year secondary patency rates for the three groups were 88%, 85%, and 84% respectively, with no statistical difference between the three groups. Endovascular treatment was chosen for high-risk patients, with favorable anatomy, proximal and distal necks of 1.5 cm, and at least on patent distal vessel. In conclusion, ET for PAA is subject to anatomic limitations related to movement of the artery, but is not inferior to surgical treatment with prosthetic grafts. It reduces the length of the patient’s hospital stay and blood transfusion requirements. Great saphenous vein grafts were not associated with better results than prosthetic grafts. However, the study recognized the need for controlled and randomized trials with appropriate long-term outcomes and inclusion criteria to compare the three treatment options. 

 In 2015, Cervin et al. 19 published the retrospective register Sweedvasc (Swedish Vascular Registry), which collected data from 30 hospitals from 2008 to 2012, analyzing 592 PAA treatments. These treatments were analyzed by emergency cases (174 with acute ischemia, 13 with rupture) and elective cases (300 asymptomatic, 105 with other symptoms). Among patients with acute ischemia, 1-year primary and secondary patency were, respectively, 78.8% and 86.8% for OT and 42.9% and 47.6% for ET. Among the symptomatic patients, 1-year primary and secondary patency were, respectively, 81.1% and 86.5% for OT and 57.1% and 85.7% for ET. Among the asymptomatic patients, 1-year primary and secondary patency rates were, respectively, 89% and 93.5% for OT and 67.4% and 83.7% for ET. 

 This study revealed an important clinical difference between the OT and ET groups, in favor of OT. Although this finding had been expected, the magnitude of the difference in results was unexpected, in particular between those treated for acute ischemia, and raises questions about use of ET to treat PAA. 

 Braga et al. 13 reported 5 years’ experience at the Hospital das Clínicas de Ribeirão Preto. Endovascular treatment using Viabahn® covered stents (a mean of 1.6 stents per patient) was chosen for patients at high surgical risk, with a minimum of two patent distal vessels, and proximal and distal necks of at least 1 cm. Open treatment was selected for patients with acute ischemia, limiting claudication, or asymptomatic patients with diameters > 2 cm, and was achieved by construction of a femoropopliteal by-pass using the great saphenous vein inverted, with distal and proximal ligature of the aneurysm. 

 A total of 10 ET and 21 OT were performed in 28 patients. There were no statistical differences in mean time in hospital or limb salvage rates at 30 or 90 days. Reintervention was necessary within 30 days in 10% of the endovascular cases and 14.28% of the open surgery group. Among the OT cases, in addition to no specific anatomical criteria for treatment, patients with critical ischemia were also treated, leading to a selection bias that affects the results and prevents intergroup comparison of the results. Therefore, prospective and controlled studies are needed to evaluate ET as an alternative procedure in cases with high risk and favorable anatomy. 

 In 2015, Von Stumm et al. 20 published a meta-analysis analyzing one randomized controlled study, one prospective cohort study, and four retrospective cohort studies, totaling 652 cases. This meta-analysis suggests that endovascular treatment for PAA may be a safe and effective method for patients with favorable anatomy. Medium-term primary patency did not differ between ET and OT, but 30-day reintervention and thrombosis rates were better in the ET group. Currently, the quality of evidence on ET is low, and additional research is absolutely necessary to support evidence-based recommendations for ET. 

 In 2017, Leake et al. 21 conducted a meta-analysis of 14 studies published from 2005 to 2016, with a total of 4,880 PAA, 3,915 managed with OT and 1,210 with ET. Patients’ comorbidities were similar in both treatment groups. Wound complications were five times more frequent after OT. Length of hospital stay was significantly shorter for ET. One and 3-year primary patency were better after OT. There were no significant differences in 1 or 3-year secondary patency. Endovascular treatment had lower rates of complications and shorter hospital stays, but primary patency was lower. Additional studies are needed to reveal long-term results. 

 In 2014, the Cochrane Collaboration published a systematic review comparing the two types of treatment for PAA. 22 Only one randomized controlled study that met the inclusion criteria was identified. That publication described 30 PAA that were treated (15 with OT and 15 with ET) and followed for at least 4 years. The mean hospital stay (4.3 days for ET and 7.7 days for OT) and duration of surgery (75.4 minutes for ET and 195.3 minutes for OT) were both significantly shorter in the ET group. One-year primary patency was 100% after OT and 93.3% after ET, but 4-year patency rates were similar. The study’s greatest limitation was the low number of cases. Currently there is no clear evidence to suggest that one or other type of elective treatment offers superior results. There is a need for more randomized trials with large enough samples to determine whether there is any advantage to using endovascular treatment rather than open treatment for PAA. 

 Although open surgical treatment is the most accepted method for PAA repair, ET is growing in popularity and interest in it is increasing, because it is less complicated and less aggressive than surgical treatment. As a result, a large number of articles have been published recently. 25 ^,^ 26 ^,^ 29 ^-^ 34

 However, to date, only reports covering short duration hospital admissions of patients treated with endovascular techniques have been published in the few articles comparing the two treatments. 29 ^-^ 34 These studies have several limitations: they are non- randomized retrospective studies, with large discrepancies between the OT and ET groups in terms of patients’ clinical presentations, short follow-up periods, and small samples, particularly in the ET group. 

 The limited number of patients covered in these comparisons prevented us from drawing consistent conclusions. New controlled and randomized studies, with long-term results and inclusion criteria that are appropriate to allow comparisons between the three treatment options are needed to enable us to determine the true indications and contraindications for endovascular treatment and its efficacy and safety. 

 The OVERPAR study 23 (“Open vs. Endovascular Repair of Popliteal Artery Aneurysm”) is a prospective, randomized, and multicenter trial that is ongoing and promises to be the largest study conducted to date in patients with asymptomatic popliteal artery aneurysms. This comparative study will provide level 1 evidence to guide treatment of patients with PAA. 

## CONCLUSIONS

 Surgical open treatment is considered the most traditional method for treatment symptomatic or asymptomatic PAA larger than 2 cm and has good rates of patency. In turn, ET initially emerged as an alternative treatment to open surgery for patients with high surgical risk, but as the years have passed it has been suggested as the first treatment option. However, there is no clear evidence that the results of one or another elective treatment are superior, as demonstrated by the Cochrane systematic review, which has a moderate evidence level. New randomized studies, with adequate samples, long follow-up periods, and well-defined criteria should be conducted to determine the role of ET in PAA.
